# ^26^Al/^10^Be Burial Dating of the Middle Pleistocene Yiyuan Hominin Fossil Site, Shandong Province, Northern China

**DOI:** 10.1038/s41598-019-43401-5

**Published:** 2019-05-06

**Authors:** Yun Guo, Chengkai Sun, Lan Luo, Linlin Yang, Fei Han, Hua Tu, Zhongping Lai, Hongchen Jiang, Christopher J. Bae, Guanjun Shen, Darryl Granger

**Affiliations:** 1 State Key Laboratory of Biogeology and Environmental Geology, School of Earth Sciences, China University of Geosciences, Wuhan, 430074 China; 2 Shandong Museum, Jinan, 250014 China; 3 School of History and Culture, Shandong University, Jinan, 250100 China; 4 State Key Laboratory of Earthquake Dynamics, Institute of Geology, China Earthquake Administration, Beijing, 100029 China; 5 Institute of Marine Science, Shantou University, Shantou, 515063 China; 6 Department of Anthropology, University of Hawai’i at Manoa, 2424 Maile Way, 346 Saunders Hall, Honolulu, HI 96822 USA; 7 College of Geographical Sciences, Nanjing Normal University, Nanjing, 210023 China; 8 Department of Earth, Atmospheric, and Planetary Sciences, Purdue University, West Lafayette, Indiana 47907 USA

**Keywords:** Evolutionary ecology, Archaeology, Geochemistry

## Abstract

The Yiyuan hominin fossil site is one of the few localities in China where a partial skullcap and several loose teeth of *Homo erectus* have been discovered. Yiyuan was previously assigned broadly to the Middle Pleistocene by biostratigraphical correlation and ESR/U-series dating. Here, we report the first application of a radio-isotopic dating method to the site. ^26^Al/^10^Be burial dating results derived from two sand samples from the fossiliferous deposits show that the hominin fossils can be confidently dated to 0.64 ± 0.08 Ma (million years ago). The reliability of this age is supported by the zero age of modern fluvial sediment near the cave. Our result is consistent with the age estimation based on biostratigraphic correlation and supports the argument that the Yiyuan and Zhoukoudian Locality 1 *H. erectus* fossils are contemporaneous. The results presented here, along with other recent chronological studies on Chinese Middle Pleistocene hominin sites, indicate that the time span from 600–400 ka (thousand years ago) is a critical period for human evolution in East Asia. Importantly, this time bracket includes several major climatic changes that would have influenced hominins, both morphologically and behaviorally.

## Introduction

The emergence of anatomically modern humans in eastern Asia continues to be a highly important topic within paleoanthropology^1,2^. Recently, evidence has been presented that suggests early modern humans were present in this region as early as 120 ka (refs^3–8^). However, the evolutionary history of the Middle Pleistocene hominin lineages preceding the appearance of *Homo sapiens* in eastern Asia remains controversial^9–16^. Taxonomically, Middle Pleistocene hominin fossils in eastern Asia that cannot be easily assigned to *Homo erectus* or to anatomically modern *H. sapiens* are usually referred to as archaic *H. sapiens* or mid-Pleistocene *Homo* (MPH) (see reviews in refs^13,17^). Thus, the nature of human evolution in the region involving *H. erectus*, MPH, and early modern humans continues to be a subject of debate.

In order to help resolve some of these outstanding debates, a systematic and reliable chronological framework for the eastern Asian, particularly Chinese, Middle Pleistocene hominin fossils is critical. With the increasing application of some well-established dating methods (e.g., U-series dating on speleothems, luminescence dating on quartz and feldspar) in recent decades the chronology of these MPH sites in China is becoming much more refined^18–23^. Unfortunately, for almost all *H. erectus* sites, accurate and precise age control has been a challenge for decades. This is because *H. erectus* sites are usually beyond the maximum age range of luminescence dating (<300 ka), and except for some rare cases (e.g., the upper layers of Zhoukoudian locality 1, where flowstone layers suitable to U-series dating could be found^24^), most of these sites are either too old for this dating method (i.e., >600 ka), or devoid of suitable speleothem formations for dating.

Fortunately, ^26^Al/^10^Be burial dating has proven to be a reliable method to date quartz sediment buried between ~0.3 Ma and ~5 Ma^25–27^. This method has been used to date several Chinese Middle Pleistocene sites including Zhoukoudian locality 1 (Lower layers)^28^ and Bailong Cave^29^, and is expected to play an increasingly important role in refining the chronological framework of *H. erectus* in eastern Asia. Here, we report the results of ^26^Al/^10^Be burial dating on the Yiyuan hominin site, an important Middle Pleistocene *H. erectus* site in China.

**Figure 1:**
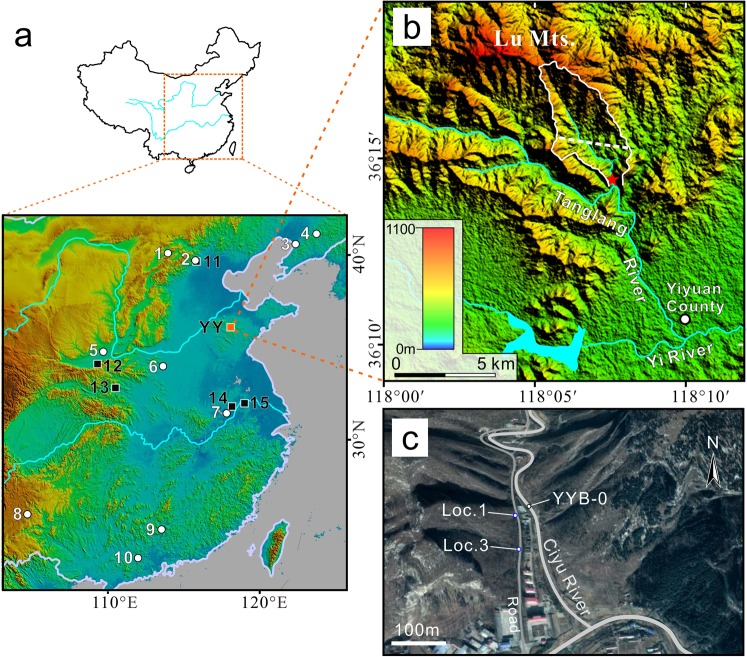
Geographical location of the Yiyuan hominin site. (**a**) Location of Yiyuan and some other contemptuous Mid-Pleistocene *Homo* (circles) and *H. erectus* (squares) sites in China, the sites are: 1. Xujiayao; 2. Zhoukoudian locality 4; 3. Jinniushan; 4. Miaohoushan; 5. Dali; 6. Lingjing; 7. Chaoxian; 8. Panxian Dadong; 9. Maba; 10. Panlong Cave; 11. Zhoukoudian locality 1; 12. Chenjiawo (Lantian); 13. Bailong Cave; 14. Hexian; 15. Tangshan. (**b**) Topographic map of the adjacent region of Yiyuan site, the white solid line marks the boundary of the drainage area of Ciyu River, the dashed line indicates a boundary between different base rock outcrop zone in the drainage area, the northern part is Paleoproterozoic granite while the southern part is Ordovician limestone. (**c**) Image showing the location of two human fossil-bearing localities of Yiyuan site, and the sampling position of YYB-0. Maps in a and b were generated using Global Mapper (www.bluemarblegeo.com) based on free ASTER GDEM v2 worldwide elevation data (http://glcf.umd.edu/data/aster/), image in c was from Google Earth & Digital Globe.

The Yiyuan hominin site (36°14′26.247″N, 118°07′35.041″E, 352 m asl.), located in Yiyuan County, Shandong Province, Northern China, consists of three small fissures/cavities developed in the Ordovician Limestone of Qizianshan (Saddle Hills) (Fig. 1). The three localities are distributed within 80 m along the right bank of the seasonal Ciyu River, about 4.5–6 m above the current riverbed. The site was first discovered during road construction, with three excavation seasons subsequently conducted in 1981–1982. All of these localities contained vertebrate fossils, with a total of 13 taxa identified (Table 1). Importantly, a fragmented hominin skullcap and two hominin teeth were discovered in locality 1 and another five hominin teeth were discovered from Locality 3 (ref.^30^). The skullcap includes parts of the parietals, frontal, and occipital bones. According to the initial morphological analysis the Yiyuan fossils were considered to be intermediate between the better known *H. erectus* fossils from Zhoukoudian and Hexian^17,30^. More recent analyses on the Yiyuan hominin teeth found that they were characterized by the presence of interproximal grooves which may be caused by habitual tooth-picking behavior^31^, and morphologically, these teeth form a coherent group together with specimens from Zhoukoudian Locality 1 and Hexian from a dental perspective^32^. Although a taphonomic analysis of the faunal assemblage has yet to be conducted, the absence of any hominin behavioral traces (e.g., Paleolithic stone tools, hearths, fire-cracked rock, etc.) and the presence of various carnivores suggests a natural accumulation.

**Table 1:** List of mammalian fauna in Yiyuan site.

**Primates**
Homo erectus*
Macaca robustus*
**Rodentia**
*Trogontherium* sp.
**Carnivora**
Canis lupus variabilis*
*Ursus arctos*
*Ursus thibetanus*
*Hyaena* sp.
Panthera tigris*
**Perissodactyla**
Stephanorhinus kirchbergensis*
Equus sanmeniensis*
**Artiodactyla**
Sus lydekkeri*
Megaloceros pachyosteus*
*Cervus* sp.
Bovinae indet.

Data collected from refs^30,34^. *Global or regional extinct taxon; sp.: taxon identified at generic level only; indet.: taxon identified at family level or above.

The Yiyuan site was first assigned to the Middle Pleistocene based on faunal correlations between Yiyuan and Zhoukoudian Loc.1 (refs^30,33^). A more recent study^34^ suggested that the extinction rate of the Yiyuan fauna is higher than that of the Chenjiawo site in Lantian (paleomagnetically dated to 0.65 Ma)^35^ and the upper layers of Zhoukoudian Loc. 1 (>400 ka by U-series dating on flowstones)^24^, but this difference may simply be because of the relatively small number of taxa recovered from Yiyuan and Chenjiawo^34^. Regardless, care should be used with assigning specific numerical ages bases specifically on biostratigraphy^8^. The first numeric ages for Yiyuan were from coupled ESR/U-series dating on nine mammalian fossil teeth from Locality 1 and 3, which resulted in ages ranging from 338 ± 25 ka to 471 ± 72 ka (ref.^36^).

**Figure 2:**
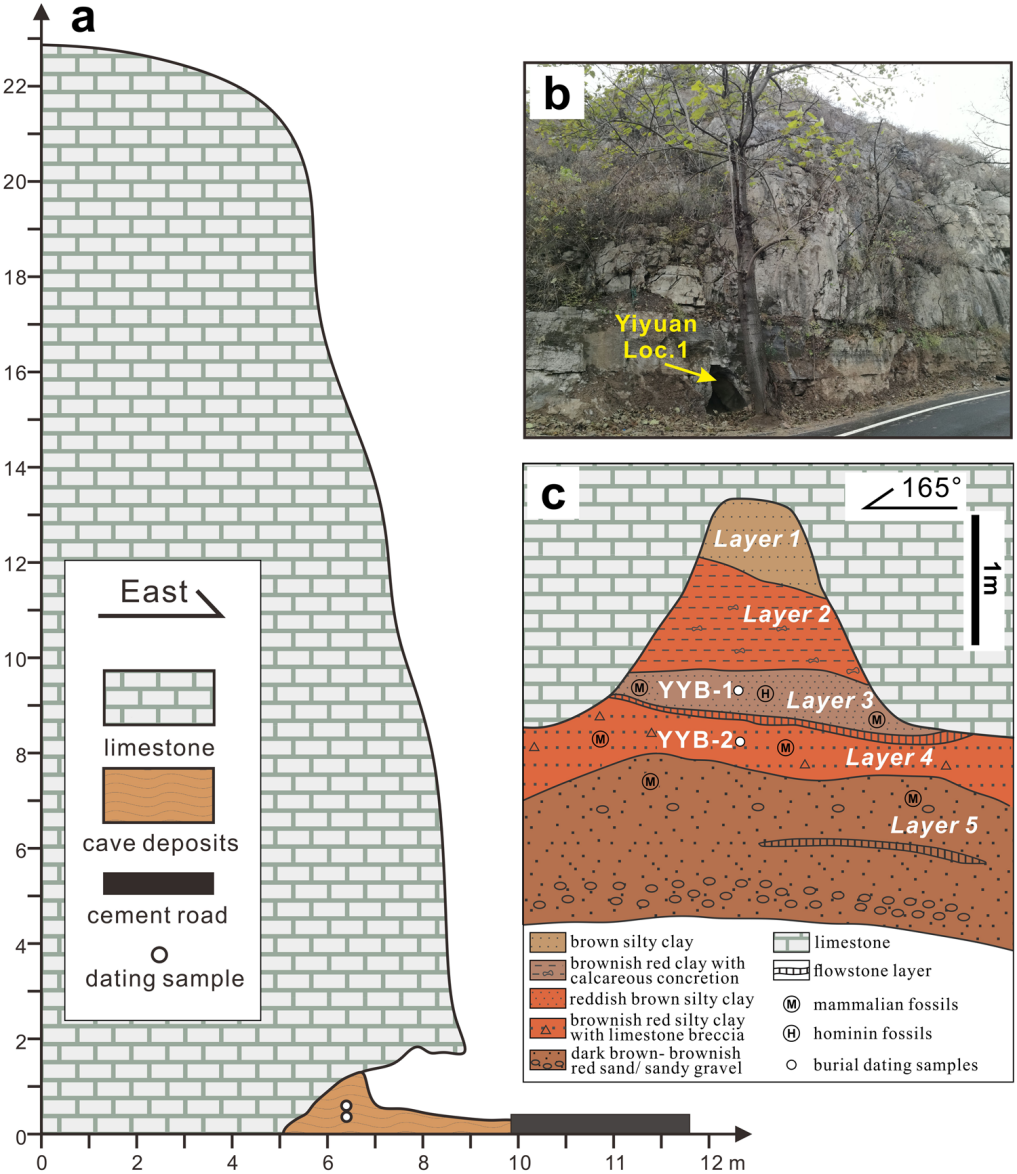
Stratigraphy and sampling information of Yiyuan Locality 1. (**a**) Sketch cross-section depicting the shielding condition of the dating samples. As shown, the samples were ~2.5 m deep from the current cave entrance and were vertically shielded by >21 m of limestone (distance measured by portable laser range finder). (**b**) A photograph taken in front of the cave entrance by one of the authors (H.T.). (**c**) Stratigraphy and sampling spots of Yiyuan Locality 1 (modified after ref.^32^).

The stratigraphic sequence of the deposits from the three Yiyuan localities are generally consistent^30^. Taking locality 1 as an example, the stratigraphy can be divided into five stratigraphic units except for the topsoil (Fig. 2c). All of the hominin fossils and a majority of the mammalian fossils were recovered from Layer 3. Mammalian fossils are less abundant in Layer 4, and sporadic in the upper part of Layer 5. Layer 1 and Layer 2 are sterile.

The majority of the Localities 2 and 3 of the Yiyuan site was destroyed during the road construction, and for Locality 1, most of the cave deposits were removed during the excavation or buried when the cement road was laid down in the 1980s. Currently, the most accessible cross section of the site is the remaining part of Locality 1, where only sediments from Layer 4 to Layer 1 are exposed. For the cosmogenic burial dating analysis, we collected several kilograms of sediment from Layer 3 (YYB-1) and Layer 4 (YYB-2) where coarse quartz grains could be extracted (Fig. 2). To test the complexity of the exposure-burial history of these cave deposits, another sample (YYB-0) was taken from the current dry riverbed of Ciyu River in front of the cave (Fig. 1c).

## Results

The ^26^Al and ^10^Be nuclide concentrations of the Yiyuan samples are presented in Table 2. Calculated simple burial ages for YYB-1 from layer 3 is 0.53 ± 0.11 Ma and for YYB-2 from layer 4 is 0.57 ± 0.07 Ma.

**Table 2:** Nuclide concentrations and burial ages of the samples from Yiyuan hominin site.

Sample ID	Description	[^26^Al] (10^6^ atoms/g)	[^10^Be] (10^6^ atoms/g)	^26^Al/^10^Be ratio	Simple burial age (Ma)	Corrected age (Ma)
YYB-0	Modern river sediment	0.6661 ± 0.0193	0.0979 ± 0.0021	6.805 ± 0.245	0.00 ± 0.07	—
YYB-1	Layer 3	0.4655 ± 0.0219	0.0895 ± 0.0021*	5.201 ± 0.273	0.53 ± 0.11	0.59 ± 0.15
YYB-2	Layer 4	0.4501 ± 0.0116	0.0885 ± 0.0019	5.086 ± 0.169	0.57 ± 0.07	0.66 ± 0.09

*Weighted mean of measurements on two aliquots of the same quartz sample (0.0911 ± 0.0028 and 0.0875 ± 0.0031).

As described in the Methods section, the accuracy of ages produced by the simple burial method relies closely on the validity of the assumptions that the sample was buried only once during the past ~10 Ma and the burial depth is deep enough so that post-burial nuclide production could be neglected. To test the first assumption, we analyzed the possible source of the cave deposits by hydrological analysis based on Digital Elevation Model data of the region. We outlined the drainage area of Ciyu River (Fig. 1b), which is the source region of the Yiyuan cave deposits. Local geological survey data suggests that the quartz mineral most likely originated in the northern part of the valley, i.e., the south piedmont of the Lu Mountains where Paleoproterozoic granite is exposed (Fig. 1b). According to the topography of the valley, there are no terraces or small basins in the drainage region, and the sediment was unlikely to be buried for a significantly long period in the upstream of the Ciyu River. In addition, the ^26^Al/^10^Be concentration ratio of the modern river sediment sample (YYB-0) corresponds well with the surface nuclide production rate ratio of 6.8 (Table 2). This suggests, with a fair degree of confidence, that we can exclude the possibility that the sediment of the Ciyu River experienced multiple burial events.

To test the second assumption mentioned above, we estimate the cosmic ray shielding condition of the cave deposits. The hillslope where Yiyuan Locality 1 situates is nearly vertical, and the cave deposits were vertically covered by more than 21 m of limestone (Fig. 2a), which is generally thick enough to shield against secondary cosmic rays^37,38^. But horizontally, the sampling cross section is currently only 2.5 m deep from the present entrance of the cave (Fig. 2a). This amount of rock is sufficient to fully shield the sample from secondary cosmic ray neutrons, but a small flux of more deeply penetrating muons may influence the calculated burial age. We estimate the muon flux by assuming that the samples are buried 20 m vertically and 2.5 m horizontally in a vertical wall of limestone. Using a three-exponent approximation for muogenic production rates^39^ and adapting the shielding calculations of Dunne *et al*.^40^ for a slope of 90°, we estimate a post-burial production rate of 0.014 atoms ^10^Be per year. For a post-burial ^26^Al/^10^Be production rate ratio of 7.0, this increases the burial ages for YYB-1 and YYB-2 to 0.59 ± 0.15 Ma and 0.66 ± 0.09 Ma respectively. The weighted average age, corrected for post-burial production, is 0.64 ± 0.08 Ma.

Additionally, although reworking of cave deposits can cause burial dating to overestimate the true age, this is unlikely for the fossiliferous deposits in Yiyuan Locality 1. The cave is quite small with only one chamber, and so there are no older sediments in higher chambers to remobilize. Moreover, sediment in the modern river has a burial age of zero as expected, indicating that quartz washed into the cave should have had no prior burial history.

## Discussion

Simple burial dating of two samples from Yiyuan Locality 1 suggests that the mammalian fossil bearing layer 4 should be older than 0.57 ± 0.07 Ma and the hominin fossil-bearing layer 3 is older than 0.53 ± 0.11 Ma. These ages provide a minimal control of the site as the samples were buried insufficiently deep to shield against cosmic rays. A correction for post-burial nuclide production resulted in ages of 0.66 ± 0.09 Ma and 0.59 ± 0.15 Ma, respectively. Because these two ages fall within the error range (±1σ) of each other, we consider their weighted mean value of 0.64 ± 0.08 Ma to best represent the age of the fauna and hominin fossils from Yiyuan.

Our results are the first radio-isotopic and reliable age control for the Yiyuan *H. erectus*. The new chronometric age generally agrees with the age estimate based on biostratigraphic correlation^30,34^. Further, these findings lend support to the hypothesis that the Yiyuan *H. erectus* is contemporaneous with Zhoukoudian Locality 1, which has stratified dates between 0.4~0.8 Ma by U-series dating on speleothem and cosmogenic burial dating^24,28^. The results presented here are somewhat older than the 420–320 ka age range previously dated by ESR/U-series dating^36^. We notice that the fossil teeth used for the ESR/U-series dating were excavated from layer 3 during the 1980s but without coordinates recorded. This makes the reconstruction of the external dose rate of the samples difficult, and thus a value recently measured by a portable gamma spectrometer from the remaining deposits was used as an alternative^36^. As Gamma dose rate may vary between different areas of a cave’s deposits simply based on factors such as difference in density of fossils and distance to limestone cave walls, it may be possible that the ESR/U-series ages are underestimated due to a biased external gamma dose rate.

The application of ^26^Al/^10^Be burial dating at the Yiyuan site is one further step toward establishing a reliable chronological framework for Middle Pleistocene *H. erectus* in eastern Asia, particularly China. Combined with the updated results of chronological studies from other Middle Pleistocene hominin fossil sites in China (Table 3), it is clear that MPH sites generally fall in the range of 500–200 ka and *H. erectus* are mostly older than 600 ka. Some *H. erectus* deposits may be a bit younger though as evidence from the upper layers of Zhoukoudian locality 1 (ref.^24^) and Hexian suggest^41^. This chronological pattern implies that the transition from *H. erectus* to MPH (or the replacement of the former by the latter) in eastern Asia most likely occurred between 600–400 ka (refs^19,21^). The evolutionary history of *H. erectus* and MPH in this region remains a point of discussion^13,15,42^.

**Table 3:** A summary on the chronology of some Middle Pleistocene hominin fossil sites from China.

Site name	Hominin fossil	Age (Ma)	Dating method	Reference
Xujiayao	mid-Pleistocene *Homo*	0.16~0.22	post-IR IRSL	Li *et al*.^55^
		0.24 ± 0.05	^26^Al/^10^Be burial	Tu *et al*.^56^
Zhoukoudian Loc. 4	mid-Pleistocene *Homo*	>0.27	U-Series	Shen *et al*.^23^
Jinniushan	mid-Pleistocene *Homo*	>0.20	ESR & U-series	Chen *et al*.^48^
Miaohoushan	mid-Pleistocene *Homo*	>0.50	U-Series	Zhang *et al*.^49^
Dali	mid-Pleistocene *Homo*	0.26–0.27	post-IR IRSL	Sun *et al*.^20^
Chaoxian	mid-Pleistocene *Homo*	0.31~0.36	U-Series	Shen *et al*.^18^
Panxian Dadong	mid-Pleistocene *Homo*	0.13~0.20 0.19~0.30	U-Series OSL	Shen *et al*.^57^ Zhang *et al*.^22^
Maba	mid-Pleistocene *Homo*	>0.28	U-Series	Shen *et al*.^19^ Xiao *et al*.^58^
Panlong Cave	mid-Pleistocene *Homo*	>0.44	U-Series	Tu *et al*.^21^
Zhoukoudian Loc. 1	*H. erectus*			
*layer 3*		>0.40	U-Series	Shen *et al*.^24^
*layer 7–10*		0.77 ± 0.08	^26^Al/^10^Be burial	Shen *et al*.^28^
Chenjiawo (Lantian)	*H. erectus*	~0.65	Paleomagnetism	An and Ho^35^
Bailong Cave	*H. erectus*	<0.76 ± 0.06 Early Brunhes 0.51 ± 0.02	^26^Al/^10^Be burial Paleomagnetism ESR/U-series	Liu *et al*.^29^ Kong *et al*.^59^ Han *et al*.^60^
Hexian	*H. erectus*	0.41 ± 0.03 ka	ESR/U-series	Grün *et al*.^41^
Tangshan	*H. erectus*	>0.58	U-Series	Zhao *et al*.^61^
Yiyuan	*H. erectus*	0.63 ± 0.06	^26^Al/^10^Be burial	This paper

In eastern Asia, evidence of hominin occupation during the Early Pleistocene is sparse, but widely distributed. Representative examples are the fossils from Gongwangling^43,44^ and Yuanmou^45^ in central and southern China (see review in ref.^15^) and lithic evidence from the high latitude Nihewan Basin in northern China^46,47^. During the middle Middle Pleistocene (typically 600–400 ka), sites bearing *H. erectus* fossils were mostly restricted to the adjacent regions of the north China plain and the middle-lower Yangtze River plains (Fig. 1a). On the other hand, MPH were much more widespread, reaching as far northeast as the Jinniushan^48^ and Miaohoushan^49^ sites and as far south as the Panlong cave site^21^ which lies near the Tropic of Cancer (Fig. 1a). One possible explanation for the change in spatial distribution patterns of *H. erectus* from the Early to Middle Pleistocene is a fundamental change in the climate system called the Middle Pleistocene transition (MPT), which occurred between ~0.9 and ~0.6 Ma (refs^50,51^). The MPT event was characterized by the shift of dominant periodicity of the glacial cycles from 40 ka to 100 ka and by the increasing climate contrast between glacial and interglacial periods^51^. It is possible that *Homo erectus* may have been able to survive variable habitats and changing climates during the Early Pleistocene, but the prolonged and intensified glacial period after MPT would have been an unprecedented challenge to this species. After the MPT, MPH may have been better able to adapt to a range of different environments, likely facilitated by biological and cultural developments including the ability to regularly procure large game^13,15,52^. The profound change in the climate system thus may have been one of the most important driving factors for Middle Pleistocene human evolution in eastern Asia and warrants further hypothesis testing.

## Methods

The burial dating method utilized here is described in detail by Granger and Muzikar^26^ and Granger^38^. To summarize, ^26^Al/^10^Be burial dating assumes that quartz mineral was gradually exposed from a steadily eroding outcrop and regularly acquired certain amounts of cosmogenic ^26^Al and ^10^Be. The mineral was then deeply buried in river terraces or caves, for which the production of cosmogenic nuclides drastically decelerates. The radioactive decay of inherited ^26^Al and ^10^Be in the buried quartz provides a means to calculate the age of said burial event. The simple burial dating method relies on two major assumptions: (1) the sample was derived from a landform of steady-state erosion prior to burial and only experienced a single burial event; and (2) the sample was buried rapidly and deeply. For the Yiyuan site we estimate a post-burial production rate by muons that contributes to the calculated burial age, as discussed in the Results Section. It should be noted that our estimation of the muon production rate is approximate, given that the equations used from Dunne *et al*.^40^ are derived for neutrons, which have a slightly different angular distribution than muons, and that we used a multi-exponential approximation for the muon flux. These differences, however, should have a negligible effect on the calculated burial ages.

For sample pre-treatment, the raw samples were rinsed by water to remove silt and clay, then soaked in HCl to remove carbonates and phosphate components. Grains between 0.2 mm and 0.5 mm were sieved out for quartz extraction through a series of treatments including HF etching and magnetic and gravimetric separation. The purified quartz was then analyzed in PRIME Lab, Purdue University. 50–100 grams of quartz for each sample was dissolved in HF/HNO_3_ and spiked with ~0.3 mg ^9^Be carrier (with 1041 ± 8 ppm of ^9^Be). No ^27^Al carrier was added for the Yiyuan samples as their natural ^27^Al concentrations are high enough for isotopic analysis. ~2 ml of aliquot was taken from the dissolved samples for Al determination by ICP-OES. After HF volatilization and pH-controlled precipitation (to remove iron and other impurities), aluminum and beryllium were separated from the sample by ion exchange chromatography, and further purified respectively. Al and Be were then precipitated as hydroxides and transformed into oxides using a propane flame. Al_2_O_3_ and BeO were then mixed with niobium and loaded into cathodes for the ^10^Be/^9^Be and ^26^Al/^27^Al measurements using the Accelerator Mass Spectrometer at the PRIME Lab in Purdue University, against standards prepared by K. Nishiizumi^53,54^. Burial calculations were made assuming steady-state erosion prior to burial, with a ^10^Be production rate of 5.54 at/g/yr and a ^26^Al/^10^Be production rate ratio of 6.8, calculated for a latitude of 36° and an elevation of 340 m. Reported uncertainties include analytical errors only, and do not include uncertainty in the production rate ratio or the radioactive decay constants.

## Data Availability

The datasets generated and/or analyzed during the current study are available from the corresponding authors on request.
